# Cerebrovascular pulsatility following long duration spaceflight is associated with changes in pulse pressure and carotid artery stiffness

**DOI:** 10.1113/EP092272

**Published:** 2025-05-19

**Authors:** Roxanne Fournier, Danielle K. Greaves, J. Kevin Shoemaker, Philippe Arbeille, Richard L. Hughson, Andrew D. Robertson

**Affiliations:** ^1^ Schlegel‐UW Research Institute for Aging Waterloo Ontario Canada; ^2^ School of Kinesiology Western University London Ontario Canada; ^3^ CERCOM‐UMPS Faculté de Médecine, Université de Tours Tours France; ^4^ Department of Kinesiology and Health Sciences University of Waterloo Waterloo Ontario Canada

**Keywords:** arterial stiffness, blood pressure, cerebral blood flow, microgravity

## Abstract

Central artery stiffening increases the haemodynamic pulsations transmitted downstream towards target organs, including the brain. While recent evidence suggests that long duration spaceflight is associated with reduced common carotid artery (CCA) distensibility, cerebrovascular pulsatility has not been extensively characterized in astronauts. This study investigated changes in pulsatility from pre‐flight to after 6 months in space, using a secondary analysis of data from four separate experiments. Middle cerebral artery blood velocity (MCAv) was measured during supine rest in 27 astronauts (20 men, 7 women). In subsets of this cohort, we measured CCA distensibility and β stiffness (*n* = 20), and CCA wave intensity (*n* = 12). The overall increase in MCAv pulsatility index (PI_mca_) from pre‐flight to post‐flight was not significant (0.73 ± 0.12 vs. 0.77 ± 0.11, *P =* 0.060, partial η^2^ = 0.13). However, individual changes in PI_mca_ were directly associated with changes in estimated aortic pulse pressure (*r* = 0.51, *P* = 0.007) and β stiffness (*r* = 0.54, *P* = 0.015), and inversely associated with changes in distensibility (*r* = −0.62, *P =* 0.003), in separate bivariate analyses. Wave intensity analysis suggested a reduction in normalized wave reflection (*P* = 0.07), and that forward compression wave amplitude was directly related to PI_mca_ (*r* = 0.64, *P =* 0.025). These findings suggest that PI_mca_ in the days immediately following spaceflight is a function of lower carotid distensibility, highlighting the interplay between arterial stiffness and cerebrovascular pulsatility.

## Abstract (highlights)

- **What is the central question of this study?**
  What are the effects of spaceflight on cerebrovascular pulsatility and its relationship to carotid distensibility, wave intensity and critical closing pressure?
- **What is the main finding and its importance?**
  While the overall effect was modest, we show that middle cerebral artery pulsatility index in the acute period following 6 months of spaceflight was elevated in some astronauts by an equivalent rate of 5–10 years of ageing on Earth. Changes were related to pulse pressure and carotid artery stiffness. These results point to potential impacts of spaceflight on brain health.

## 1 INTRODUCTION

Spaceflight exposes astronauts to multiple arterial stressors, including radiation, social changes, sleep disturbances and altered nutrition (Barratt et al., 2019). One of the unique characteristics of spaceflight is weightlessness (i.e. microgravity), which is now acknowledged as a significant contributor to vascular changes in astronauts. Specifically, long‐duration exposure to microgravity on the International Space Station (ISS) is associated with a shift of fluid towards the neck and head (Arbeille et al., 2015), and vascular alterations suggestive of central artery stiffening (Baevsky et al., 2007; Hughson et al., 2016). The cephalad fluid shift is believed to result in cerebral venous congestion and altered venous emptying (Arbeille et al., 2021; Marshall‐Goebel et al., 2019), which may contribute to increased cerebral blood volume/congestion and altered haemodynamics. Arterial stiffening increases pulse pressure and leads to the propagation of cardiac‐related haemodynamics downstream towards target organs, including the brain (Mitchell et al., 2011; O'Rourke & Hashimoto, 2007). Over the past decade, an increasing amount of evidence has linked arterial haemodynamic pulsatility to adverse changes in brain health, including elevated risk for cognitive impairment and stroke (Chiesa et al., 2019; Mitchell et al., 2011; Ott et al., 2013; Shi et al., 2020; Webb et al., 2022). Pulsatility more closely relates to early deleterious changes in cerebrovascular health than quantitative cerebral blood flow (Shi et al., 2020). Whether cerebrovascular pulsatility is increased in astronauts on their return from spaceflight has not been established.

Evidence for altered cerebrovascular regulation following long‐duration (i.e. ∼6 months) spaceflight is mixed. Across studies, post‐flight middle cerebral artery (MCA) mean blood velocity (MCAv_mean_) was unchanged (Tobal et al., 2001; Zuj et al., 2012), decreased (Arbeille et al., 2001) or increased (Iwasaki et al., 2021) compared to pre‐flight measures. Furthermore, dynamic cerebrovascular autoregulation was impaired (Zuj et al., 2012) or preserved/enhanced (Iwasaki et al., 2007; Tobal et al., 2001), and cerebrovascular reactivity to carbon dioxide was impaired (Zuj et al., 2012). Cerebrovascular pulsatility in astronauts is rarely reported and evidence of changes during or following long‐duration ISS missions from limited sample sizes has been equivocal (Arbeille et al., 2001; Tobal et al., 2001; Zuj et al., 2012).

In healthy individuals, cerebrovascular pulsatility, as assessed by Gosling's pulsatility index (PI), can be interpreted as a function of mean arterial pressure (MAP), pulse pressure (PP) and back pressure (Gosling et al., 1991). Back pressure is akin to critical closing pressure (CrCP), a functional indicator of cerebrovascular regulation related to the combined effect of intracranial pressure (ICP) and arteriolar wall tension (Panerai, 2003), as well as vascular impedance (Michel & Zernikow, 1998). Thus, pulsatility is influenced by central arterial blood pressure regulation, damping of pressure by elastic components of central arteries, and local variables associated with cerebral congestion and the contractile state of vascular smooth muscle. An alternative method to quantify pulsatile energy propagated along the vasculature is wave intensity analysis of concordant blood velocity and pressure waveforms (Bleasdale et al., 2003). Wave intensity characteristics obtained from the common carotid artery (CCA) are associated with cardiac contractility, aortic damping (Sugawara et al., 2009), and reflections that are related to cerebrovascular tone (Bleasdale et al., 2003). Pulsatility in the MCA (PI_mca_) has been directly related to CCA forward wave intensity, while pulsatile damping between the CCA and MCA has been related to the ratio of backward to forward wave intensities (Lefferts et al., 2020).

Women now represent 50% of newly recruited astronauts, and research efforts are advocating for greater understanding of sex‐ and gender‐specific responses to spaceflight to optimize their health and safety (Platts et al., 2014). Female astronauts have been recognized as being more susceptible to post‐flight orthostatic intolerance, with a greater reduction in plasma volume being posited as one mechanism (Waters et al., 2002). To date, sex differences in cerebrovascular responses to spaceflight have not been explored; however, women are known to have lower pulsatile damping compared to men (Lefferts et al., 2020). Women also have greater age‐associated increases in carotid stiffness and carotid pulse pressure, while men have greater age‐associated increases in aortic stiffness (Lefferts et al., 2023). These factors likely contribute to greater age‐associated increases in PI_mca_ in women (Alwatban et al., 2021; Lefferts et al., 2020, 2023), pointing to the possible existence of sex‐specific mechanisms modulating PI_mca_ (Lefferts et al., 2023).

Through a secondary analysis of previously collected data across four studies from astronauts before and after 6 months in space, we aimed to investigate the effects of spaceflight on cerebrovascular pulsatility through the lens of changes in CCA distensibility, wave intensity, and CrCP. We hypothesized that cerebrovascular pulsatility would be increased following spaceflight. We posited that this increase would be associated with reduced CCA distensibility and increased forward wave intensity, and that CrCP would also be elevated post‐flight, indicative of increased cerebrovascular tone. Furthermore, we leveraged a larger sample, from multiple studies, to perform an exploratory analysis on how sex modulates these relationships.

## 2 METHODS

### 2.1 Ethical approval

This secondary analysis of anonymous research data was approved by the University of Waterloo Human Ethics Board (ORE#41976; 26/02/2020). Each of the individual studies (see below) had previously received ethics clearance from review boards at the University of Waterloo, the National Aeronautics and Space Administration, the European Space Agency, and the Japanese Space Agency. All procedures conformed to the *Declaration of Helsinki* (2013), other than prior registration in a database, and written, informed consent was obtained from each participant prior to testing.

### 2.2 Study participants and design

We amalgamated data that were collected as part of four separate studies: Cardiovascular and Cerebrovascular Control on Return from the International Space Station (CCISS) (*n* = 7, 1 woman; 2007–2010), Vascular (*n* = 9, 4 women; 2009–2013), Vascular Echo (*n* = 10, 2 women; 2015–2023), and Vascular Aging (*n* = 4, 1 woman; 2017–2023). We have reported partial data from CCISS (Zuj et al., 2012) and Vascular (Hughson et al., 2016) previously. Each study was a repeated measures observational design. We acquired pre‐flight data 66 ± 30 days before launch and post‐flight data within 4 days of landing: Recovery Day 0 for CCISS, Recovery Day 1 for Vascular, and Recovery Day 4 for Vascular Echo and Vascular Aging. The average flight duration was 169 ± 32 days. No astronauts were repeat participants across the four studies.

### 2.3 Data acquisition

We obtained physiological measures while participants were lying supine after at least 10 min of rest. Across the four studies, we acquired data using similar methods but with varying devices/configurations. We acquired ECG using a lead‐II configuration. We acquired arterial blood pressure using the volume‐clamp technique on the left middle finger, and we estimated brachial and aortic pressure waveforms using generalized transfer functions with Beatscope software (Finapres Medical Systems B.V., Enschede, the Netherlands). We calibrated brachial waveforms to the average of two consecutive manual pressure measurements obtained using a sphygmomanometer and stethoscope on the contralateral arm. We then calibrated the aortic pressure waveform to the mean and diastolic values of the calibrated brachial waveform. We also used the finger pressure waveform to estimate the left ventricular outflow using the Modelflow algorithm (Finapres). We acquired MCAv from the left cerebral hemisphere using transcranial Doppler ultrasound. In most cases, we acquired these physiological signals simultaneously using a single acquisition system (PowerLab, ADInstruments, Colorado Springs, CO, USA). In 13 cases, we acquired MCAv separately and time‐synced the signal to physiological data using a simultaneous data‐tag. We visually confirmed all data sets and removed sequences with transient signal loss; 50 of 54 datasets (i.e. pre and post datasets from 27 participants) included sequences longer than 10 cardiac cycles.

In the Vascular series studies (i.e. Vascular, Vascular Echo, and Vascular Aging), we measured right side CCA blood velocity (*n* = 12, 3 women) and arterial diameter (*n* = 20, 7 women) using ultrasound, from which wave intensity and distensibility analyses were performed. We took measurements >1 cm proximal to the carotid bifurcation. We quantified blood velocity as the peak spectral trace from Doppler velocity imaging (i.e. outer envelope; MAUI, Hedgehog Medical, Waterloo, Ontario Canada) over at least five cardiac cycles. We measured diastolic and systolic CCA diameters on M‐mode imaging, using digital calipers placed on the intima‐lumen border at the smallest and largest excursions of the vessel wall across a cardiac cycle over at least 5 cycles. Concurrent with imaging, we acquired contralateral (i.e. left) CCA pressure waveforms using applanation tonometry (SPT‐301, Millar, Houston, TX, USA) and calibrated these against simultaneous mean and diastolic brachial artery pressures (Kelly & Fitchett, 1992).

### 2.4 Data analysis

We extracted the maximum, minimum and mean of arterial blood pressure and MCAv beat‐by‐beat gated to the ECG R‐spike, corresponding to systolic, diastolic, and mean values, respectively. We calculated PP as systolic minus diastolic blood pressure, heart rate as 60/RR‐interval, stroke volume as the integral of the estimated left ventricular outflow, and cardiac output as the product of heart rate and stroke volume. In addition, we calculated total peripheral resistance as MAP divided by cardiac output, and total arterial compliance as stroke volume divided by PP. We calculated cerebrovascular conductance index as MCAv_mean_ divided by MAP, and cerebrovascular resistance index as the inverse of conductance. Site specific variables, herein, are denoted by subscripting with MCA, CCA, ba (brachial) and aorta.

#### 2.4.1 Haemodynamic pulsatility

We calculated PI using Gosling's method as the amplitude of the blood velocity waveform normalized to the mean (Gosling et al., 1991). We then quantified the damping efficiency as the ratio of haemodynamic pulsatility between carotid and cerebral arteries (i.e. PI_cca_/PI_mca_), with a larger number indicating greater damping (Zarrinkoob et al., 2016).

#### 2.4.2 Critical closing pressure

We estimated CrCP and resistance area product using the first harmonic method (Panerai et al., 2011). First, we determined the amplitude of the first harmonic of each of the brachial artery blood pressure and MCAv waveforms (P_1_ and V_1_, respectively) using fast Fourier transform. Then, we calculated resistance area product as the ratio of the amplitudes (i.e. *P*_1_/*V*_1_), and CrCP as

$$\mathrm{CrCP}=P_{0}\;-V_{0}\times \left(P_{1}/V_{1}\right)$$

where *P*_0_ and *V*_0_ reflect mean blood pressure and velocity, respectively (Panerai et al., 2011).

#### 2.4.3 Carotid artery stiffness

We calculated CCA distensibility coefficient (cDC) and β stiffness index from simultaneous pressure and diameter measurements, as previously described (Hughson et al., 2016):

$$\mathrm{cDC}=\left({D_{s}}^{2}-{D_{d}}^{2}\right)/\left(\mathrm{PP}\times {D_{d}}^{2}\right)$$

$$\beta \;\mathrm{stiffness}\;\mathrm{index}=\left[\ln\left(P_{s}/P_{d}\right)\right]/\left[\left(D_{s}-D_{d}\right)/D_{d}\right]$$

where *D*_s_ is systolic diameter, *D*_d_ is diastolic diameter, PP is arterial pulse pressure, *P*_s_ is systolic pressure and *P*_d_ is diastolic pressure. We calculated cDC in terms of both PP_ba_ and PP_cca_ (cDC_ba_ and cDC_cca_, respectively) and β stiffness in terms of PP_cca_.

#### 2.4.4 Wave intensity analysis

We performed wave intensity analysis using the product of the first derivatives of the CCA blood pressure and velocity waveforms (Sugawara et al., 2009). Briefly, we first extracted pressure and velocity waveforms from foot‐to‐foot using the tangent to the second derivative to identify the waveform feet. We normalized each waveform to the cardiac cycle and ensemble averaged the waveforms within each session. We then confirmed waveform alignment by examining the linear relationship between pressure and velocity during the initial systolic upslope (i.e. approximately the first 50 ms, representing a period free of wave reflection (Zambanini et al., 2005)). We performed waveform separation to distinguish forward and reflected waveforms, and then calculated wave intensity for forward traveling waves and backward traveling waves, separately (Mynard et al., 2020). From the forward traveling waves, two wave intensity characteristics were defined: a forward compression wave during early systole indicated by increasing pressure and velocity, and a forward expansion wave during late systole indicated by decreasing pressure and velocity. From the backward traveling waves, a negative area marks a backward compression wave during mid systole indicated by increasing pressure and decreasing velocity. We calculated a reflection index as the ratio of backward to forward compression waves.

### 2.5 Statistics

We used R (version 4.3.3) with RStudio for statistical analysis and plotting (R Core Team, 2023). We estimated the effect of spaceflight on the haemodynamic and vascular characteristics described above using linear mixed‐effects models, incorporating session (pre‐flight, post‐flight) as a fixed‐effect factor and participant as a random‐effect factor to account for repeated measures and random intercept. To confirm that the results were not confounded by differences in measurement devices between studies, a sensitivity analysis was performed that included study as an additional random effect. Subsequently, we assessed the association of PI_mca_ with a variety of central/CCA and cerebrovascular characteristics using two steps. First, we used partial correlation to test the relationship between post‐flight PI_mca_ and the change in variables of interest while controlling for pre‐flight PI_mca_. We corrected for multiple comparisons in the correlational analysis using false discovery rate. To visualize associations highlighted by this partial correlation step, we plotted the change in PI_mca_ vs. the change in the vascular characteristic. Secondly, based on the outcome of the partial correlation, we used a linear mixed‐effects model to assess the association between PI_mca_ and PP_aorta_, while controlling for cerebrovascular resistance and heart rate – two known modulators of haemodynamic pulsatility (de Riva et al., 2012). Specifically, PP_aorta_, resistance area product and heart rate were included as fixed effects, and participant was included as a random effect to allow different intercepts between astronauts. An a priori type I error rate was set at α = 0.05, and partial eta squared (η^2^) effect sizes and 95% confidence intervals were calculated to support statistical inferences. All model fits were evaluated by examining quantile plots for residuals and random effects, testing normality of residuals using the Shapiro–Wilk test, and assessing multicollinearity between predictor variables using the variance inflation factor. Finally, we performed an exploratory analysis on the role of sex in our findings. With respect to the influence of spaceflight, we added a sex × spaceflight interaction fixed effect to our original model. With respect to the association between PI_mca_ and PP_aorta_, we included the sex × PP interaction term. Given the relatively small sample being investigated, we report only the partial η^2^ effect sizes from this exploratory work on sex effects.

## 3 RESULTS

From the combined sample of 30 astronauts, we excluded three participants (1 woman) from the final analysis: one due to a change in medication during the study, one due to a technical issue during data acquisition, and one due to research constraints during the COVID‐19 pandemic that prevented post‐flight measurements until 14 days after landing. Table 1 shows cardiovascular haemodynamics measured during pre‐ and post‐flight assessments in the final sample of 27 astronauts. Each of systolic, diastolic, mean and pulse pressure from the brachial artery was elevated post‐flight. Systolic pressure showed the largest effect size, being the primary driver of changes in mean and pulse pressure. An increase in PP_aorta_ was more muted, although the effect size was still moderate. Heart rate was also elevated after spaceflight. In contrast, modelled estimates of stroke volume and cardiac output were unchanged with negligible effect sizes. Estimates of total peripheral resistance and arterial compliance were also unchanged post‐flight, although a moderate effect towards decreased compliance was noted.

**TABLE 1:** Cardiovascular hemodynamic characteristics pre‐ and post‐flight.

Variable	Pre‐flight, *n* = 27	Post‐flight, *n* = 27	*P*	Partial η^2^ (95% CI)
Brachial artery blood pressure				
Systolic pressure (mmHg)	115 (14)	126 (14)	**<0.001**	**0.37 (0.09, 0.59)**
Diastolic pressure (mmHg)	70 (10)	74 (11)	**0.03**	**0.18 (0.00, 0.43)**
Mean pressure (mmHg)	87 (11)	93 (12)	**0.003**	**0.30 (0.05, 0.53)**
Pulse pressure (mmHg)	45 (10)	51 (11)	**0.04**	**0.16 (0.00, 0.41)**
Pulse pressure – aorta (mmHg)	35 (7)	38 (9)	0.09	0.10 (0.00, 0.35)
Heart rate (beat min^−1^)	56 (10)	59 (9)	**0.005**	**0.27 (0.03, 0.51)**
Stroke volume (mL)	80 (18)	79 (16)	0.82	0.00 (0.00, 0.12)
Cardiac output (L min^−1^)	4.5 (1.0)	4.7 (1.2)	0.39	0.03 (0.00, 0.24)
Total peripheral resistance (mmHg min L^−1^)	20.9 (6.3)	21.2 (5.8)	0.75	0.00 (0.00, 0.16)
Total arterial compliance (mL mmHg^−1^)	2.4 (0.8)	2.1 (0.5)	0.10	0.10 (0.00, 0.35)

Data are presented as means (SD). *P*‐value and effect size (partial η^2^) were estimated using linear mixed‐effects modelling with a fixed effect of spaceflight and random effect of participant. Significant fixed effects are shown in bold. CI, confidence interval.

### 3.1 Haemodynamic pulsatility

**FIGURE 1:**
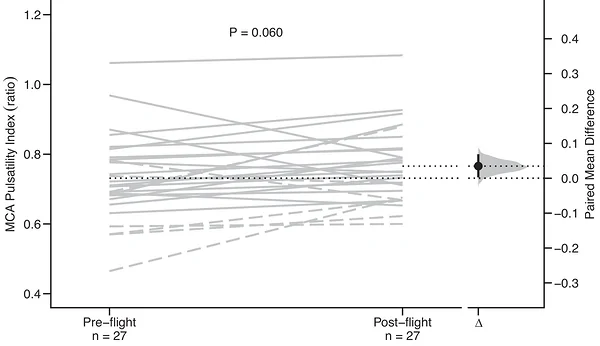
The change in middle cerebral artery (MCA) haemodynamic pulsatility (PI_mca_) from pre‐ to post‐flight. The left side of the figure shows responses of individual male (continuous lines; *n* = 20) and female (dashed lines; *n* = 7) astronauts. The horizontal black dotted lines spanning the figure reflect the group mean during pre‐ and post‐flight assessments. The *P*‐value represents significance of the fixed effect of spaceflight using linear mixed‐effect regression modelling. The right side of the figure shows a visualization of the paired mean difference effect size using bootstrapped resampling (1000 resamples). A mean difference of zero is aligned to the pre‐flight mean value. The bootstrapped distribution of mean pre–post differences is shown in grey. The data point and error bars reflect the mean effect and 95% confidence interval (i.e. over 95% of the bootstrapped distribution shows a positive mean difference, indicative of an increase in PI_mca_ post‐flight).

MCA haemodynamic characteristics, including CrCP, were largely unchanged post‐flight with negligible effect sizes (Table 2). A notable exception was PI_mca_, which tended to be elevated post‐flight with a moderate effect size despite individual variability (Figure 1). In the subset of participants in whom CCA pulsed‐wave Doppler imaging was acquired (Vascular Echo and Vascular Aging, *n* = 12, Table 3), PI_cca_ was unchanged post‐flight, though we note that PI_mca_ in this subset was similarly unchanged (mean ΔPI_mca_ = +0.01). The pulsatile damping ratio between CCA and MCA was also unchanged (Table 3).

**TABLE 2:** Middle cerebral artery haemodynamic characteristics pre‐ and post‐flight.

Variable	Pre‐flight, *n* = 27	Post‐flight, *n* = 27	*P*	Partial η^2^ (95% CI)
Systolic blood velocity (cm s^−1^)	80 (23)	82 (26)	0.82	0.00 (0.00, 0.13)
Diastolic blood velocity (cm s^−1^)	40 (14)	39 (12)	0.57	0.01 (0.00, 0.19)
Mean blood velocity (cm s^−1^)	57 (18)	56 (18)	0.84	0.00 (0.00, 0.12)
Pulsatility index (ratio)	0.73 (0.12)	0.77 (0.11)	0.06	0.13 (0.00, 0.38)
Vascular conductance (cm s^−1^ mmHg^−1^)	0.67 (0.29)	0.61 (0.21)	0.23	0.05 (0.00, 0.28)
Vascular resistance (mmHg s cm^−1^)	1.73 (0.75)	1.94 (1.01)	0.23	0.05 (0.00, 0.28)
Resistance area product (mmHg s cm^−1^)	1.28 (0.58)	1.44 (0.70)	0.20	0.06 (0.00, 0.29)
Critical closing pressure (mmHg)	9 (9)	7 (7)	0.67	0.00 (0.00, 0.17)

Data are presented as means (SD). *P*‐value and effect size (partial η^2^) were estimated using linear mixed‐effects modelling with a fixed effect of spaceflight and random effect of participant. CI, confidence interval.

**TABLE 3:** Common carotid artery haemodynamic characteristics pre‐ and post‐flight.

Variable	Pre‐flight, *n* = 20	Post‐flight, *n* = 20	*P*	Partial η^2^ (95% CI)
Pulse pressure (mmHg)	43 (13)	47 (11)	0.09	0.14 (0.00, 0.43)
Systolic diameter (mm)	6.06 (0.62)	6.12 (0.69)	0.53	0.02 (0.00, 0.26)
Diastolic diameter (mm)	5.45 (0.61)	5.55 (0.62)	0.34	0.05 (0.00, 0.31)
Pulse diameter (mm)	0.60 (0.15)	0.57 (0.15)	0.27	0.06 (0.00, 0.34)
Distensibility (brachial PP) (mmHg^−1^ × 10^−3^)	4.9 (1.6)	3.9 (1.1)	**0.01**	**0.28 (0.02, 0.55)**
Distensibility (carotid PP) (mmHg^−1^ × 10^−3^)	5.8 (2.3)	4.7 (1.5)	**0.03**	**0.23 (0.00, 0.51)**
Beta stiffness (arbitrary units	4.7 (1.5)	5.4 (1.6)	**0.04**	**0.21 (0.00, 0.49)**
Systolic blood velocity (cm s^−1^) (*n* = 12)	98 (12)	93 (18)	0.19	0.15 (0.00, 0.51)
Diastolic blood velocity (cm s^−1^) (*n* = 12)	26 (2)	24 (5)	0.26	0.06 (0.00, 0.31)
Mean blood velocity (cm s^−1^) (*n* = 12)	42 (4)	41 (8)	0.90	0.00 (0.00, 0.20)
Pulsatility index (ratio) (*n* = 12)	1.73 (0.23)	1.68 (0.31)	0.64	0.02 (0.00, 0.34)
Pulsatile damping (ratio) (*n* = 12)	2.28 (0.40)	2.18 (0.40)	0.29	0.09 (0.00, 0.44)
FCW amplitude (mmHg cm s^−3^) (*n* = 12)	12.46 (4.33)	14.66 (7.35)	0.13	0.19 (0.00, 0.55)
FEW amplitude (mmHg cm s^−3^) (*n* = 12)	1.55 (0.89)	1.41 (0.69)	0.46	0.05 (0.00, 0.40)
BCW amplitude (mmHg cm s^−3^) (*n* = 12)	−4.31 (1.94)	−4.35 (2.50)	0.95	0.00 (0.00, 0.10)
Reflection index (ratio) (*n* = 12)	0.41 (0.18)	0.29 (0.13)	0.07	0.14 (0.00, 0.41)

Data are presented as means (SD). *P*‐value and effect size (partial η^2^) were estimated using linear mixed‐effects modelling with a fixed effect of spaceflight and random effect of participant. Significant fixed effects are shown in bold. BCW, backward compression wave; CI, confidence interval; FCW, forward compression wave; FEW, forward expansion wave; PP, pulse pressure.

### 3.2 Carotid artery stiffness and wave intensity

All measures of carotid arterial wall stiffness were changed during post‐flight testing. Both cDC_ba_ and cDC_cca_ decreased, and β stiffness index increased, each showing large effect sizes (Table 3). The reflection index was reduced with a moderate effect size but was not significant. This apparent reduction in wave reflection was driven by an ∼18% elevation (non‐significant) of forward compression wave amplitude. The forward expansion waves and backward compression waves were unchanged post‐flight.

### 3.3 Associations between PI_mca_ and vascular characteristics

**FIGURE 2:**
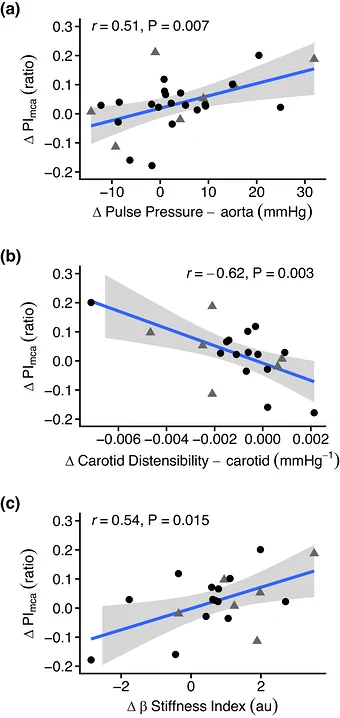
Associations between the change in middle cerebral artery pulsatility index (PI_mca_) and indices of arterial stiffness. (a) There was a direct correlation with changes in estimated aortic pulse pressure (*n* = 27). (b) There was an inverse correlation with changes in common carotid artery (CCA) distensibility (*n* = 20). (c) There was a direct correlation with changes in CCA β stiffness index (*n* = 20). The blue line and grey ribbon represent the unstandardized linear regression coefficient and standard error, respectively. Data points reflect individual male (black circles) and female (grey triangles) astronauts. Pearson's correlation coefficients and *P*‐values are reported.

**FIGURE 3:**
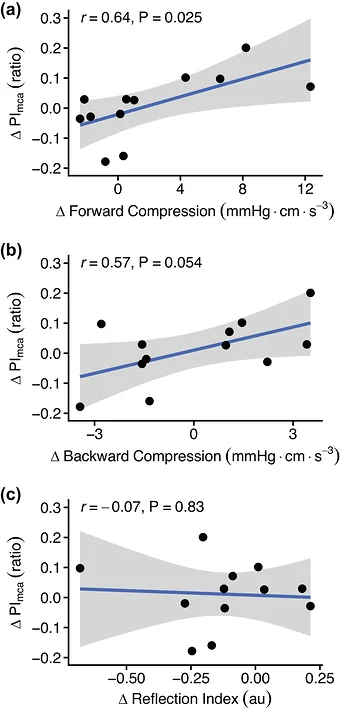
Associations between the change in middle cerebral artery pulsatility index (PI_mca_) and indices of common carotid artery wave intensity. (a) There was a direct correlation with changes in the forward compression wave. (b, c) However, there was no significant correlation with changes in backward wave compression (b) or the reflection index (ratio of backward:forward; c). The blue line and grey ribbon represent the unstandardized linear regression coefficient and standard error, respectively. Data points reflect individual astronauts (*n* = 12); male and female participants were not differentiated due to the small sample size. Pearson's correlation coefficients and *P*‐values are reported.

In partial correlation analyses, post‐flight PI_mca_ was directly related to the change in PP_aorta_ after *P*‐value adjustment for multiple comparisons (Table 4). This association between PP and PI_mca_ remained significant when substituting either PP_cca_ (*n* = 20) or PP_ba_ (*n* = 27) for the estimated aortic values (neither shown). We also observed associations between PI_mca_ and our two measures of arterial stiffness – cDC_cca_ and β stiffness index (unadjusted *P*‐values < 0.04). Further evidence to support these associations is observed in the relationships between the change in PI_mca_ and that of PP_aorta_, cDC_cca_ and β stiffness index, respectively (Figure 2a–c). The association between PI_mca_ and PP_aorta_ remained strong after adjusting for heart rate (HR) and resistance area product, with PP_aorta_ accounting for ∼1/3 of the variance in PI_mca_ (Table 5). In addition, a strong partial correlation between post‐flight PI_mca_ and forward compression wave amplitude was observed, but the statistical significance was limited by the smaller sample size used for wave intensity analysis (Table 4). The change in PI_mca_ was directly related to the forward compression wave (Figure 3a) and tended to be associated with the backward compression wave (Figure 3b), but not the wave intensity reflection index (Figure 3c).

**TABLE 4:** Partial correlations with post‐flight PI_mca_ after adjustment for pre‐flight PI_mca_.

Variable	*n*	*r*	*P*
Δ Heart rate	27	0.29	0.24
Δ Mean arterial pressure	27	0.06	0.85
Δ Critical closing pressure	27	−0.26	0.29
Δ Pulse pressure – aorta	27	**0.62**	**0.007**
Δ CCA diameter (diastole)	20	−0.02	0.94
Δ Distensibility (CCA pressure)	20	−0.54	0.06
Δ β stiffness	20	0.48	0.10
Δ FCW amplitude	12	0.70	0.06
Δ BCW amplitude	12	0.58	0.12
Δ Reflection index	12	0.13	0.85

Correlation coefficients (*r*) reflect partial correlation of post‐flight PI_mca_ with each variable after adjustment for pre‐flight PI_mca_. *P*‐values are adjusted for false discovery rate to account for multiple comparisons. Significant fixed effects are shown in bold. BCW, backward compression wave; CCA, common carotid artery; FCW, forward compression wave.

**TABLE 5:** Predictors of the MCA pulsatility pre/post spaceflight (*n* = 27).

	Model 1				Model 2
	*B* (95% CI) ^a^	β	*P*	Partial η^2^ (95% CI)	Partial η^2^ (95% CI)
Fixed effects					
Intercept	**0.75 (0.71, 0.78)**	**0.75**	**<0.001**		
PP_aorta_	**6.1 (3.5, 8.9)**	**0.05**	**<0.001**	**0.31 (0.10, 0.50)**	**0.30 (0.10, 0.49)**
HR	**−3.4 (−6.6, 0.0)**	**−0.03**	**0.05**	**0.10 (0.00, 0.31)**	0.05 (0.00, 0.23)
RAP	−16.8 (−59.8, 0.2)	−0.003	0.44	0.01 (0.00, 0.13)	0.03 (0.00, 0.18)
Sex (female)					**0.25 (0.02, 0.51)**
Sex × PP_aorta_					<0.01 (0.00, 0.12)
Random effects	SD				SD
Intercept (ID)	0.08				0.07
Residual	0.06				0.06

Results were tested by linear mixed‐effects modelling. Significant fixed effects are shown in bold. ^a^Unstandardized coefficients (*B*) are reported as x10^−3^ (except for intercept). CI, confidence interval; HR, heart rate; ID, participant identifier; PP, pulse pressure; RAP, resistance area product.

### 3.4 Effect of sex

The final sample included 20 men (48 ± 7 years) and 7 women (42 ± 5 years). In the exploratory analysis of sex differences, the interaction of spaceflight and sex had little effect on PI_mca_ (partial η^2^ = 0.03; 95% CI: 0, 0.23). Of note, a large main effect of sex was observed, wherein male astronauts had higher PI_mca_ than female astronauts (see line type in Figure 1; partial η^2^ = 0.26; 95% CI: 0.03, 0.50). As such, inclusion of sex in the linear model strengthened the main effect of spaceflight on PI_mca_ [*P =* 0.044, as compared to *P =* 0.06 without sex in the model (Table 2); a significant improvement in model fit (ANOVA test) was observed when including sex, *P =* 0.014]. Furthermore, no interaction effect of spaceflight and sex was observed for either PP_aorta_ or cDC_cca_; however, a medium effect size for their interaction on β stiffness index was observed suggesting that stiffness may increase more in female astronauts [*n* = 20 (6 women); partial η^2^ = 0.14; 95% CI: 0.00, 0.42]. The sample of female astronauts with CCA blood velocity measurements was too limited (*n* = 2) to support an analysis of sex with respect to wave intensity.

## 4 DISCUSSION

Amalgamation of cardio‐ and cerebrovascular data from four distinct spaceflight studies provided evidence in modest support of our hypothesis that increases in arterial stiffness following 6 months onboard the ISS were associated with elevated cerebral haemodynamic pulsatility. While the unadjusted change in PI_mca_ did not meet the a priori determined threshold for statistical inference (*P =* 0.06), several indicators support the conclusion that PI_mca_ is elevated post‐flight. First, the effect size was robust and, while this synthesis of data from four distinct studies created the largest sample of spaceflight MCAv data available, it was still limited to less than 30 participants. Second, although sex was not balanced in the cohort and thus sex‐related findings were interpreted with caution, adjustment for sex resulted in a statistically significant effect of spaceflight on PI_mca_. Finally, a strong association between PI_mca_ and each of PP_aorta_, cDC_cca_ and β stiffness index was noted. A significant association was also found between PI_mca_ and the forward compression wave. Contrary to one hypothesis, CrCP was not elevated post‐flight. We found that PI_mca_ was higher in men than women but the hypothesized greater increase in PI_mca_ in women was not supported, possibly due to the slightly younger age of the women. Together, these data point to potential increases in the pulsatile burden to the brain that could have long‐term health consequences related to risk for stroke or cognitive impairment (Chiesa et al., 2019; Mitchell et al., 2011; Ott et al., 2013; Shi et al., 2020; Webb et al., 2022) for astronauts exposed to microgravity and the space environment.

On average, PI_mca_ increased from pre‐flight to post‐flight by ∼0.03. Although small, the confidence in this observation was strengthened when controlling for sex (Alwatban et al., 2021; Lefferts et al., 2023). Prior literature has reported no main effects of spaceflight on PI_mca_ or Pourcelot's resistive index (Tobal et al., 2001; Zuj et al., 2012); however, these previous studies were limited by sample sizes of six or seven astronauts/cosmonauts, and by statistical models which tested the cerebrovascular response to lower body negative pressure, confounding simple spaceflight effects. Between the third and fifth decade of life – in line with the age of our astronaut sample – PI_mca_ has been reported to increase at a mean rate between 0.003 and 0.006 units/year (Alwatban et al., 2021). When characterizing PI changes across the lifespan, non‐linear increases in PI appear to be present after 40 years of age (Lefferts et al., 2020, 2023; Tarumi et al., 2014), with age–sex interactions potentially contributing to higher age‐adjusted PI in women (Alwatban et al., 2021; Lefferts et al., 2023). Thus, the increase in PI_mca_ observed after 6 months of spaceflight might be as high as 5–10 years of ageing, on average, though this ‘ageing effect’ is likely to be age‐ and sex‐dependent. Previously, using a subset of the current dataset, we compared changes in carotid artery stiffness following 6 months in space to that which occurs over 10–20 years on Earth (Hughson et al., 2016). While this evidence of increased cerebrovascular pulsatility fits with the notion that spaceflight is associated with ‘accelerated vascular ageing’, the absence of change in other cerebrovascular regulatory variables, namely conductance, resistance and CrCP, suggests that local cerebral regulatory function may either be maintained or recover rapidly in the days immediately after spaceflight. Increased pulsatility at the MCA following 6 months in space may thus be a direct result of increased stiffness in more proximal vessels, such as the aorta and carotid artery. It remains unresolved as to whether longer spaceflight missions, as well as those beyond low‐Earth orbit, may challenge these cerebrovascular regulatory mechanisms beyond their capacity to modulate the potential deleterious effects of central artery stiffness and downstream pulsatility. In‐flight data may help to resolve the proposed links between central and cerebrovascular effects.

Cross‐sectional and modelling studies have linked stiffening of the aorta and large extracranial arteries, as well as a widening of pulse pressure, to greater wave transmission toward the brain, resulting in increased carotid and cerebral blood flow pulsatility (Lefferts et al., 2023; Mitchell et al., 2011; Mynard et al., 2017). In line with those findings, we observed an inverse association between PI_mca_ and cDC, and a direct association with CCA β stiffness. The β stiffness index is a robust marker of central artery stiffness that is less dependent on variations in MAP compared to the cDC (Morioka et al., 2021). Since we observed an increase in MAP post‐flight, the changes in the cDC may simply indicate a new set point around which the functional characteristics of the CCA wall are operating. However, the parallel associations between PI_mca_ and β stiffness, as well as the absence of any change in CCA dimensions, strengthen our assertion of a post‐flight increase in arterial stiffness.

In the subset of 12 astronauts for whom we had CCA Doppler blood velocity waveforms, wave intensity analysis showed a strong effect for an increased forward compression wave post‐flight, with no change in the backward compression wave amplitude. As such, we calculated a reflection index that tended to be lower post‐flight, suggestive of more pulsatile energy being transmitted into the cerebral circulation. The forward compression wave is a measure of energy propagation in early systole resulting from left ventricular ejection. Some cardiovascular adaptation is perhaps expected due to reduced blood volume (Alfrey et al., 1996) and lower cardiac demands during everyday activities (Fraser et al., 2012) while in‐flight. Recent data, however, show that cardiac mass, volumes and ejection fraction are maintained following long duration spaceflight (Shibata et al., 2023) suggesting that cardiac function is preserved following spaceflight, although with individual variability. No conclusions can be made regarding the aetiology of increased wave intensity in the current study; however, we posit that regional differences in stiffening between the aorta and carotid may have contributed to increased pressure transmission without changes in stroke volume. Interestingly, systolic blood velocities were unchanged from pre‐ to post‐flight, and we found no change in PI_cca_, or in vascular damping between the CCA and MCA. Instead, overall changes in PI_mca_ appeared to be a function of slight, non‐significant decreases in diastolic and mean blood velocities more so than increased systolic velocities.

The individual variability in responses prompts a closer look at the role of CrCP, which on average was unaffected by spaceflight. PI_mca_ can be derived from the ratio of PP and the difference between MAP and CrCP (Michel & Zernikow, 1998), where CrCP reflects the back pressure (Czosnyka et al., 1996). We calculated CrCP using a well‐described method (Panerai et al., 2011); however, this calculation is based on arterial dynamics alone, and CrCP, in theory, is subject to the combined influence of vascular tone and ICP. ICP has not been measured directly during spaceflight but has been measured from Ommaya reservoirs during brief periods of microgravity during parabolic flight (Lawley et al., 2017). ICP was reduced during microgravity relative to lying supine, but remained slightly elevated relative to upright sitting in 1G. Additionally, non‐invasive estimates of ICP in the first 3 days after spaceflight were not different from pre‐flight (Iwasaki et al., 2021), although direct lumbar puncture performed 12– 60 days after spaceflight found mild elevations of cerebrospinal fluid opening pressure, as an indicator of ICP, in two astronauts with ocular symptoms, but no elevation in two others (Mader et al., 2011). In‐flight middle cerebral vein velocity has been found to be increased relative to pre‐flight supine levels, alongside a decreased luminal diameter, which acutely recovered when applying lower‐body negative pressure (Arbeille et al., 2021). This may point to increased transmural pressure on cerebral vessels, and possibly mild brain tamponade caused by ICP. Direct or indirect measurements of ICP in‐flight may enhance our understanding of changes in CrCP in astronauts, and its implications for the regulation of haemodynamic pulsatility during spaceflight.

Potential sex differences in the effects of spaceflight on cerebrovascular pulsatility were investigated because women have lower pulsatile damping and greater age‐associated increases in carotid stiffness and pulse pressure (Alwatban et al., 2021; Lefferts et al., 2020, 2023). Men had higher PI_mca_ than women (Figure 1), and the exploratory inclusion of sex in the linear model strengthened the main effect of spaceflight on PI_mca_. However, no interaction effect of sex on spaceflight was noted. The women in the study were younger than the men, which may have lessened the expected change in PI_mca_ with spaceflight. Furthermore, given the range of ages amongst the female astronauts (34–50 years), some may have been at various stages of the menopause transition. Female hormones have been shown to be protective against increases in PI_mca_ (Penotti et al., 1996), and thus differences in hormone levels (not measured in the current studies) may confound our interpretation. As more women enter the astronaut corps, and as active female astronauts continue to age, future research can achieve better sex balance and explore pulsatility and other cardio‐ and cerebrovascular characteristics in greater detail.

### 4.1 Limitations

Spaceflight research is typically limited to small sample sizes due to the restriction in access to astronauts with their responsibilities to multiple research projects and daily crew activities. By combining data from four different research projects that had many measurements in common as part of the overall investigations of vascular health, we were able to increase this sample size for the main outcome of PI_mca_. One consequence of this synthesis is that data were collected over a 16‐year period. During that time, astronaut exercise routines, nutrition and work schedules have evolved substantially. Astronauts engage in their preferred types, intensities and durations of exercise (Fraser et al., 2012; Hughson et al., 2016), and the exercise hardware is periodically upgraded (e.g. the interim resistive exercise device (iRED) was replaced with the advanced resistive exercise device (ARED) in 2009); they select their preferred meals from a standardized menu and are permitted to bring additional specialty foods (Smith et al., 2019); and frequency/duration of high‐stress extravehicular activities varies within each crewmember and over time. These variable factors interact over time with normal human variation to influence the study outcomes. In addition, as noted, data were collected with different equipment over the four studies aggregated within this report. The measures and outcomes of each individual study also differed to some degree resulting in admittedly smaller sample sizes for the distensibility and wave intensity analyses. Thus, these data should be interpreted with caution but can be beneficial to planning of future research. The strong linear relationships between variables measured with independent devices point to the strong physiological effects of spaceflight on arterial properties and cerebrovascular health consequences.

In addition to these broad caveats, a few methodological assumptions deserve consideration. First, while no Earth‐based control group was used as a comparator in this observational study, insonation parameters (e.g. depth, gain, sample volume) and anatomical landmarks were used to ensure replication of measurement sites pre‐ and post‐flight, and to minimize the opportunity for spurious measurement error. Second, inconsistent signals due to small insonation windows (e.g. transcranial Doppler) or instability in tonometer measurements limited the clean data to as few as five cardiac cycles for some participants, which may inaccurately represent average resting steady‐state haemodynamics. Notably, 93% of datasets were acquired over at least 10 cardiac cycles. Finally, the logistics of data collection necessitated that we obtain CCA ultrasound‐based metrics on the right side, and MCAv on the left. Despite anatomical differences between the right and left CCA in terms of branching from the aorta, we assumed that hemispheric differences would be minimal based on evidence that differences in the functional indices of PI and stiffness between sides are negligible (Hernández et al., 2003; Uithoven et al., 2017).

### 4.2 Impact

The associations reported here describe steady state changes shortly after returning to Earth. The presence of elevated pulsatile transmission into the cerebral circulation during spaceflight still requires attention. Lee and colleagues showed the CCA was distended in‐flight, although the effect on local stiffness metrics was marginal (Lee et al., 2020). While coincident blood pressure changes in that study were equivocal, pressures are often reported as being reduced in‐flight – especially during diastole – consistent with reduced peripheral vascular resistance (Arbeille et al., 2001; Baevsky et al., 2007; Norsk et al., 2015). The reduction in arterial pressure in space appears contrary to observations of elevated cardiac output relative to pre‐flight upright seated rest (Hughson et al., 2017; Norsk et al., 2015), as well as the higher muscle sympathetic nerve activity recorded during short‐duration space shuttle missions (Ertl et al., 2002). Furthermore, acute fluctuations in arterial pressure may lead to distinct responses in cerebrovascular pulsatility (e.g. acute hypotension; Moir et al., 2020) which are not explored here. Collectively, the impact of these changes on cerebrovascular pulsatility while in space is unknown. In‐flight PI_mca_ has not been reported previously but one study of seven cosmonauts showed no change from pre‐flight between 2 weeks and 6 months of spaceflight in the cerebrovascular resistivity index – an alternate measure of normalized pulsatile haemodynamics (Arbeille et al., 2001). Continued study of the relationship between in‐flight changes in fluid distribution, pressure and cerebrovascular haemodynamics, especially as humans explore beyond low‐Earth orbit into the high galactic cosmic radiation environment with its negative consequences for endothelial and vascular health (Hughson et al., 2018), will further our understanding of mechanisms related to the acute changes in pulsatility following return to Earth suggested here. Furthermore, the duration of elevated pulsatility during and post‐flight will ultimately determine the weight of any heightened risk of spaceflight on long term brain health.

### 4.3 Exercise as a countermeasure during spaceflight

Current countermeasures used in low Earth orbit spaceflight to maintain health, such as the requirements for exercise and nutrition, are targeted to combat muscle atrophy, bone loss and reduced aerobic power. While exercise opportunities on ISS are multi‐modal, including resistance and aerobic devices, training programmes often side with individual astronaut preferences. Resistance training is overall protective of brain health on Earth (Allison & Al‐Khazraji, 2024); however, it preferentially contributes to increased cerebrovascular resistance (Thomas et al., 2021) and altered cerebral autoregulation (Roy et al., 2022) compared to aerobic training. On the other hand, resistance exercise was shown to both increase (Nakamura & Muraoka, 2018) and decrease PI_mca_ (Thomas et al., 2021). Further understanding of the effects of exercise modality on cerebrovascular health in the context of arterial stiffening and cephalad fluid shifts during spaceflight is necessary to better inform recommended exercise countermeasures.

### 4.4 Conclusions

With this study, we show that cerebral haemodynamic pulsatility in the acute period following long‐duration spaceflight is tightly related to central pulse pressures and carotid artery stiffness. While individual variability was observed, we note a moderate to strong effect of spaceflight on increased carotid artery stiffness and cerebrovascular pulsatility. Long‐term elevations in pulsatility could present increased risks for stroke and damage to small cerebral vessels. Future research is needed to better examine the underlying individual variability, with important insight likely to be derived from comprehensive in‐flight behavioural and physiological monitoring. Consideration of exercise and nutritional countermeasures, and the cumulative exposure to, and possible synergistic effects between, the multiple hazards of space, including radiation, social isolation, physical confinement and poor sleep quality, are especially needed as space programmes extend the duration of missions to facilitate exploration beyond low Earth orbit.

## AUTHOR CONTRIBUTIONS

Richard Hughson and Andrew Robertson conceived and designed the secondary research design. Danielle Greaves, Kevin Shoemaker, Philippe Arbeille, Richard Hughson, and Andrew Robertson acquired the original data at Johnson Space Centre (USA), Kennedy Space Centre (USA), the European Astronaut Centre (Germany), and Star City (Russia). Roxanne Fournier and Andrew Robertson performed the secondary data extraction and analyzed the data at the Schlegel‐UW Research Institute for Aging at the University of Waterloo (Canada). Roxanne Fournier, Richard Hughson, and Andrew Robertson interpreted the data. Roxanne Fournier and Andrew Robertson prepared figures and drafted the manuscript. All authors were involved in the critical evaluation of the manuscript for intellectual content, approved the final version of the manuscript, and agree to be accountable for all aspects of the work in ensuring that questions related to the accuracy or integrity of any part of the work are appropriately investigated and resolved. All persons designated as authors qualify for authorship, and all those who qualify for authorship are listed.

## CONFLICT OF INTEREST

None declared.

## Data Availability

The data that support the findings of this study are available from the co‐author Prof. Richard Hughson (hughson@uwaterloo.ca) upon reasonable request.
